# Innate Immune Tolerance in Microglia Does Not Impact on Central Nervous System Prion Disease

**DOI:** 10.3389/fncel.2022.918883

**Published:** 2022-06-30

**Authors:** Reiss Pal, Barry M. Bradford, Neil A. Mabbott

**Affiliations:** The Roslin Institute and Royal (Dick) School of Veterinary Studies, University of Edinburgh, Easter Bush, Edinburgh, United Kingdom

**Keywords:** microglia, prion disease, central nervous system, lipopolysaccharide, innate immune tolerance

## Abstract

Prion diseases such as Creutzfeldt-Jakob disease in humans, bovine spongiform encephalopathy in cattle, and scrapie in sheep, are infectious and chronic neurodegenerative diseases to which there are no cures. Infection with prions in the central nervous system (CNS) ultimately causes extensive neurodegeneration, and this is accompanied by prominent microglial and astrocytic activation in affected regions. The microglia are the CNS macrophages and help maintain neuronal homeostasis, clear dead or dying cells and provide defense against pathogens. The microglia also provide neuroprotection during CNS prion disease, but their pro-inflammatory activation may exacerbate the development of the neuropathology. Innate immune tolerance induced by consecutive systemic bacterial lipopolysaccharide (LPS) treatment can induce long-term epigenetic changes in the microglia in the brain that several months later can dampen their responsiveness to subsequent LPS treatment and impede the development of neuritic damage in a transgenic mouse model of Alzheimer’s disease-like pathology. We therefore reasoned that innate immune tolerance in microglia might similarly impede the subsequent development of CNS prion disease. To test this hypothesis groups of mice were first infected with prions by intracerebral injection, and 35 days later given four consecutive systemic injections with LPS to induce innate immune tolerance. Our data show that consecutive systemic LPS treatment did not affect the subsequent development of CNS prion disease. Our data suggests innate immune tolerance in microglia does not influence the subsequent onset of prion disease-induced neuropathology in mice, despite previously published evidence of this effect in an Alzheimer’s disease mouse model.

## Introduction

Prion diseases (also known as transmissible spongiform encephalopathies) are sub-acute neurodegenerative diseases that affect humans and certain captive and free-ranging mammals including ovine, bovine, and cervid species. Infectious prions are considered to comprise of PrP^Sc^, abnormally folded isoforms of the host-encoded cellular prion protein, PrP^C^ (Legname et al., 2004). High levels of PrP^Sc^ accumulate in affected tissues during prion disease. Once the prions infect the central nervous system (CNS), they ultimately cause extensive neurodegeneration and vacuolar (spongiform) degeneration as well as microglial and astrocytic activation. Currently, no cures are available to treat prion-infected individuals.

The microglia are the parenchymal macrophages of the CNS and are considered to have essential roles in CNS development and homeostasis (reviewed in Prinz et al., 2019). During CNS prion disease the microglia appear to play a neuroprotective role and constrain the neurotoxic activation of reactive astrocytes (Zhu et al., 2016; Carroll et al., 2018; Bradford et al., 2021). However, the disease-associated activation of microglia can contribute to the development of neuropathology in some CNS disorders (Keren-Shaul et al., 2017), including prion diseases (Alibhai et al., 2016; Hu et al., 2021).

In the steady state, the continual production of factors derived from the commensal gut and lung microbiome plays an important role in maintaining microglial homeostasis and function (Erny et al., 2015; Hosang et al., 2022). However, ablation or modification of the commensal microbiomes at these sites can impede microglial responses to LPS treatment, infection with lymphocytic choriomeningitis virus (Erny et al., 2015), or autoimmunity in the CNS (experimental allergic encephalitis; Hosang et al., 2022). Studies have also shown how systemic inflammation or pathogen co-infection can modify the development of certain neurodegenerative disorders (Mabbott et al., 2020). In Alzheimer’s disease patients, for example, systemic inflammation coincides with increased cognitive decline (Holmes et al., 2009) and alterations to the gut microbiome might enhance the progression of the neuropathology (Xie et al., 2022). Gastrointestinal infections might similarly increase the risk of Parkinson’s disease (Nerius et al., 2020). Experimental studies have also shown how systemic LPS-mediated inflammation or gastrointestinal infections can exacerbate the progression of the clinical signs or the neuropathology in the brains of mice infected with prions (Combrinck et al., 2002; Cunningham et al., 2005; Donaldson et al., 2020) or ischemic brain damage (Denes et al., 2010).

In many of the above examples, the treatments or infections were initiated when detectable signs of neuropathology were already evident in the CNS. However, studies have shown how consecutive, systemic, LPS treatments can induce epigenetic changes in the microglia in the CNS that can persist in these cells for at least 6 months after their initial application (Wendeln et al., 2018). This epigenetic reprogramming induced innate immune tolerance in the microglia that rendered them hypo-responsive to subsequent stimulation. As a consequence, consecutive LPS treatment caused long-term changes in the microglia that months later were sufficient to alter the development of pathology in an experimental mouse model of stroke (focal cortical ischemia) and impede the subsequent development of neuropathology in a spontaneous transgenic mouse model of Alzheimer’s disease-like pathology (Wendeln et al., 2018). Other studies have shown how similar systemic stimuli (Zhou et al., 2020), a high-fat diet (Vargas-Rodríguez et al., 2022), and early life thermal stress (Ben-Nun et al., 2022) can cause long-term changes in microglial responses that can impede their responses to subsequent pro-inflammatory stimuli. The immune tolerance induced in the microglia to these repeated stimuli may be a neuroprotective response to protect the CNS from repetitive or subsequent pro-inflammatory insults (Lajqi et al., 2020; Zhou et al., 2020). Conversely, sepsis was shown to induce innate immune training in microglia that increased susceptibility to amyloid-β induced neuropathology (De Sousa et al., 2021). Although effects on microglia were not determined, mild infection with the SARS-CoV-2 coronavirus has also been shown to cause the persistent pro-inflammatory activation of monocytes and macrophages that may have long-term effects on subsequent immune responses (Bohnacker et al., 2022).

The impact of innate immune tolerance in microglia on the subsequent development of CNS prion disease was not known. Therefore, in the current study, we used a mouse model of CNS prion disease to determine whether exposure to consecutive, systemic, LPS treatments soon after prion infection would cause similar long-term changes in the microglia that months later could modulate the development of the neuropathology. A thorough understanding of the factors that modulate microglial phenotype and function during CNS prion disease may identify novel targets for therapeutic intervention in these currently untreatable neurodegenerative disorders.

## Materials and Methods

### Mice

Female C57BL/6J mice were purchased from Charles River (Margate, United Kingdom) and used throughout this study. The mice were maintained in-house under specific pathogen-free conditions and used in experiments at 6–10 weeks old. All *in vivo* mouse studies were performed under the authority of a UK Home Office Project License in accordance with the regulations of the UK Home Office “Animals (scientific procedures) Act 1986.” Approval for the individual studies was obtained after review from the University of Edinburgh’s ethical review committee.

### *In vivo* Treatment With Bacterial Lipopolysaccharide

At the times indicated mice were given daily intraperitoneal (IP) injections with bacterial LPS derived from *Salmonella enterica* serotype Typhimurium (Sigma-Aldrich, Poole, Dorset, United Kingdom) at 500 μg/kg body weight in sterile PBS. Parallel groups of mice were injected IP with 50 μL sterile PBS as control.

### Prion Infection and Clinical Disease Assessment

Groups of female C57BL/6J mice (5–6 mice/group) were injected intracerebrally (IC) into the right medial temporal lobe with 20 μL of a 1% (weight/volume) brain homogenate prepared from mice terminally infected with mouse-passaged ME7 scrapie prions. Following injection with prions, the mice were coded and assessed blindly at daily intervals for the clinical signs of prion disease. The mice were culled at a standard humane end-point upon the development of terminal clinical signs of prion disease, as described previously (Donaldson et al., 2020). Brains were removed and cut in half sagittally across the midline to separate the 2 hemispheres. One brain half was immediately flash frozen at the temperature of liquid nitrogen for gene expression or protein analysis. The other brain half was fixed in 10% neutral buffered formalin for at least 48 h prior to histopathological processing.

Clinical prion disease was confirmed by histopathological assessment of the abundance and distribution of the spongiform vacuolar degeneration in paraffin-embedded, hematoxylin and eosin (H&E) stained coronal brain sections as described previously (Fraser and Dickinson, 1967). Vacuolar lesion profiles were prepared by scoring the presence and severity of the prion disease-specific vacuolation in nine gray matter and three white matter areas using a 0–5 scale: G1, dorsal medulla; G2, cerebellar cortex; G3, superior colliculus; G4, hypothalamus; G5, thalamus; G6, hippocampus; G7, septum; G8, retrosplenial and adjacent motor cortex; G9, cingulate and adjacent motor cortex; W1, inferior and middle cerebellar peduncles; W2, decussation of superior cerebellar peduncles; and W3, cerebellar peduncles.

### Real Time-Quantitative PCR (RT-qPCR) Analysis

Snap-frozen half brains were homogenized using Lysing Matrix D tubes (MP Biomedicals, Cambridge, United Kingdom) and a Ribolyser tissue homogenizer (Bio-Rad Laboratories, Watford, United Kingdom). Total RNA was extracted using RNABee (AmsBio, Abingdon, United Kingdom), purified using an RNeasy Mini kit (Qiagen, Manchester, United Kingdom), and treated with RNase-free DNase I (Promega, Southampton, United Kingdom) to remove genomic DNA. First-strand cDNA synthesis was performed using 1 μg total RNA and the SuperScript III Reverse Transcriptase (Life Technologies, Waltham, MA, United States), and mRNA amplified using Oligo DT (Promega). RT-qPCR was then performed using the primers listed in Table 1 and FastStart Universal SYBR Green Master mix (Rox; Sigma-Aldrich) on an MX3005P RT-qPCR system (Agilent Technologies LDA UK Ltd., Stockport, Cheshire, United Kingdom). Cycle threshold values were analyzed using MxPro software (Agilent Technologies LDA UK Ltd.) and normalized relative to the reference gene *Rpl19* using the ΔΔCT method. Expression values were normalized so that the mean level in the 1xPBS-treated controls was 1.0.

**TABLE 1:** RT-qPCR primers.

Gene	Forward primer	Reverse primer
*Aif1*	GGATCAACAAGCAATTCCTCGA	CTGAGAAAGTCAGAGT AGCTGA
*Csf1r*	AGGCAGGCTGGAATAATCTGACCT	CGTCACAGAACAGGACA TCAGAGC
*Cx3cr1*	CAGCATCGACCGGTACCTT	GCTGCACTGTCCGGTTGTT
*Gbp2*	GGGGTCACTGTCTGACCACT	GGGAAACCTGGGATGAGATT
*Gfap*	AGAAAGGTTGAATCGCTGGA	CGGCGATAGTCGTTAGCTTC
*Iigp1*	GGGGCAATAGCTCATTGGTA	ACCTCGAAGACATCCCCTTT
*Il1*β	TGCCACCTTTTGACAGTGATG	TGATGTGCTGCT GCGAGATT
*Il6*	ACCAGAGGAAATTTTCAATAGGC	TGATGCACTTGCAGAAAACA
*Il10*	CCCTTTGCTATGGTGTCCTT	TGGTTTCTCTTCCCAAGACC
*Itgam*	TGGCCTATACAAGCTTGGCTTT	AAAGGCCGTTACTGAGGTGG
*Psmb8*	CAGTCCTGAAGAGGCCTACG	CACTTTCACCCAACCGTCTT
*Prnp*	TACCCTAACCAAGTGTAC TACAGGCC	TGGTACTGGGTGACGCA CATCTGCTC
*Rpl19*	GAAGGTCAAAGGGAATGTGTTCA	CCTTGTCTGCCTTCAGCTTGT
*Srgn*	GCAAGGTTATCCTGCTCGGA	TGGGAGGGCCGATGTTATTG
*Tmem119*	GTGTCTAACAGGCCCCAGAA	AGCCACGTGGTATCAAGGAG
*Tnf*	TGTGCTCAGAGCTTTCAACAA	CTTGATGGTGGTGCATGAGA

### Serum Cytokine Quantitation by ELISA

Concentrations of interleukin-1β (IL-1β), IL-6, IL-10, and tumor necrosis factor-α (TNFα) in serum samples were measured using a mouse IL-1 beta, mouse IL-6, mouse IL-10, and mouse TNF alpha uncoated ELISA kits, respectively (Thermo Fisher Scientific), according to the manufacturers’ instructions.

### Immunohistochemistry and Image Analysis

Paraffin-embedded sections (thickness 6 μm) were deparaffinized and pre-treated by autoclaving in target retrieval solution (Dako) at 121°C for 15 min. Endogenous peroxidases were quenched by immersion in 4% hydrogen peroxide in methanol for 10 min. Microglia were detected by immunostaining with rabbit anti-allograft inflammatory factor-1 (AIF1; also known as ionized calcium binding adaptor molecule 1, Iba1) polyclonal antibody (Wako, Japan) and astrocytes were detected using rabbit anti-glial fibrillary acidic protein (GFAP) polyclonal antibody (Agilent Dako). Prior to immunostaining to detect PrP, sections were immersed in 98% formic acid for 10 min. PrP was then detected by immunostaining with mouse anti-PrP monoclonal antibody BH-1 (McCutcheon et al., 2014). Following addition of biotinylated secondary antibodies, immunostaining was revealed using the Vectastain avidin-biotin complex (ABC) kit (Vector Laboratories) with 3, 3′-diaminobenzidine tetrahydrochloride (DAB) as a substrate. Sections were counterstained with hematoxylin before mounting and imaging.

Stained sections were imaged using a Brightfield Eclipse Ni-E light microscope (Nikon Instruments Europe BV, Amsterdam, Netherlands), and images were captured using Zen 2 software (Carl Zeiss Ltd., Cambridge, United Kingdom). The abundance of the AIF1 +, GFAP +, and disease-specific PrP (PrP*^d^*+) immunostaining on DAB stained sections was measured using Fiji/ImageJ software^1^ using the analyze particle algorithm as described previously (Bradford et al., 2019). Briefly, DAB-stained images of the CA1 region of the hippocampus striatum radiatum were de-convoluted, intensity thresholds applied, and mean gray OD values measured from grayscale images using a scale of 0–255. Data for each image are expressed as a proportion of the total area of pixel area analyzed (% area coverage). The morphology of AIF1 + microglia (dendrite length, number of dendrite segments, number of dendrite branch points, and number of terminal points) in the CA1 region of the hippocampus striatum radiatum of each mouse was assessed on DAB stained sections using IMARIS (version 9.5.1) software (Bitplane, Zurich, Switzerland) as previously described (Bradford et al., 2017b). Data were collected from 28 to 32 microglia/group. Individual data points are presented as mean value/mouse.

### Western Blotting

Prion-specific PrP^Sc^ was detected in flash-frozen brain samples by Western blotting as described previously (Bradford et al., 2017a). Briefly, brain homogenates (10% weight/volume) were prepared in NP40 lysis buffer [1% NP40, 0.5% sodium deoxycholate, 150 mM NaCl, 50 mM TrisHCl (pH 7.5)]. To detect relatively proteinase-resistant PrP^Sc^ a sample of the homogenate was treated with 20 μg/ml proteinase K (PK) at 37°C for 1 h. The digestion was stopped by addition of 1 mM phenylmethylsulfonyl fluoride. Samples were then denatured by incubation at 85°C for 15 min in 1X SDS sample buffer (Life Technologies) and separated via electrophoresis using 12% Tris-glycine polyacrylamide gels (Nupage, Life Technologies). Proteins were then transferred to polyvinylidene difluoride (PVDF) membranes by semi-dry electroblotting and PrP detected using mouse monoclonal antibody BH1, and β-actin detected using mouse monoclonal antibody C4 (Santa Cruz Biotechnology). Membranes were subsequently stained with horseradish peroxidase-conjugated goat anti-species specific antibody (Jackson Immunoresearch) and visualized using chemiluminescence (BM Chemiluminescent substrate kit, Roche, Burgess Hill, United Kingdom). The relative abundance of PrP in each sample on the Western blots was compared by densitometric analysis using ImageJ software. The PrP abundance in each sample was normalized to β-actin and expressed as the percentage PrP in individual samples presented relative to the mean value in the PBS-treated controls which was set at 100%.

### Statistics

Statistical significance between groups was tested using Prism 7.0 software (GraphPad, San Diego, United States). Datasets were first compared for normality using the Shapiro–Wilk normality test. Differences between groups were then compared using one-way ANOVA and *post hoc* Tukey multiple comparisons tests. Survival curve data for prion-infected mice in each treatment group were compared by Log-rank (Mantel-Cox) test. Data are expressed as dot plots of individual animal observations with median values indicated by a horizontal bar. Body weight and brain lesion profile data are presented as mean ± SD. Values of *P* < 0.05 were accepted as significant. *P < 0.05; ^**^P < 0.01; ^***^P < 0.001.

## Results

### Multiple Lipopolysaccharide Exposure Induces Innate Immune Tolerance

**FIGURE 1:**
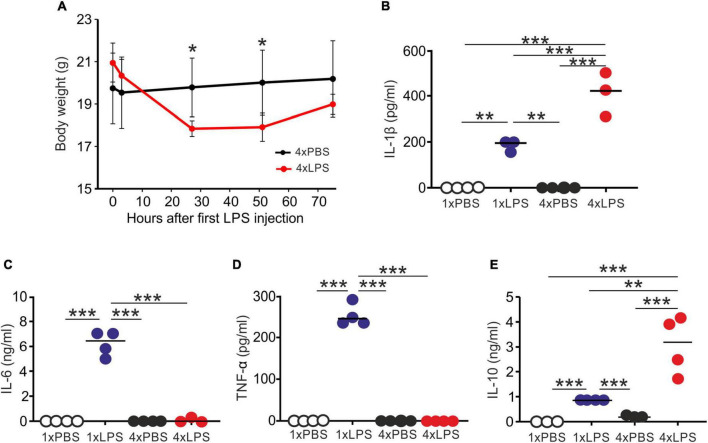
Multiple LPS exposure induces innate immune tolerance. **(A)** Groups of mice (*n* = 4/group) were given four daily IP injections with either LPS or PBS (control) and body weight measured at intervals afterward. **(B,C)** Mice were given a single (1x) or four (4x) daily IP injections with either LPS or PBS (control) and serum collected 3 h after the final injection. **(B)** Concentration of IL-1β in the serum of mice given a single or four daily IP injections with either LPS or PBS. **(C)** Concentration of IL-6 in the serum of mice given a single or four daily IP injections with either LPS or PBS. **(D)** Concentration of TNF-α in the serum of mice given a single or four daily IP injections with either LPS or PBS. **(E)** Concentration of IL-10 in the serum of mice given a single or four daily IP injections with either LPS or PBS. *N* = 3–4 mice/group; horizontal bar, median. *P < 0.05; **P < 0.01; ***P < 0.001.

**FIGURE 2:**
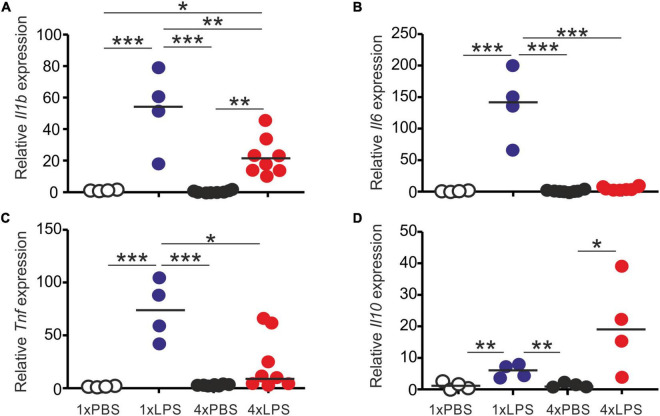
Effect of systemic LPS treatment on expression of cytokine-encoding genes in the brain. Mice were given a single (1x) or four (4x) daily IP injections with either LPS or PBS (control). Brains were collected 3 h after the final injection and gene expression compared by RT-qPCR analysis. **(A)** Relative expression level of *Il1b* mRNA. **(B)** Relative expression level of *Il6* mRNA. **(C)** Relative expression level of *Tnf* mRNA. **(D)** Relative expression level of *Il10* mRNA. Gene expression data are normalized so that the mean level in the 1xPBS controls was 1.0. *N* = 4–8 mice/group; horizontal bar, median. *P < 0.05; **P < 0.01; ***P < 0.001.

First, we compared the effects of single or multiple systemic LPS injections on cytokine production in the blood and brain. Groups of female C57BL/6J mice were treated daily with LPS by intraperitoneal (IP) injection for 1 or 4 days (1xLPS and 4xLPS, respectively) to induce innate immune training or tolerance, respectively (Wendeln et al., 2018). Parallel groups of mice were injected with PBS as a control. Brains and peripheral blood were collected 3 h after the final treatment. As anticipated, LPS treatment induced a transient period of mild sickness signs and weight loss which had begun to resolve by day 4 of treatment (Figure 1A). High levels of the pro-inflammatory cytokines interleukin-1β (IL-1β), IL-6, and tumor necrosis factor-α (TNF-α) were detected in the serum 3 h after 1 dose of LPS (Figures 1B–D). However, the levels of IL-6 and TNF-α in the serum 4xLPS-treated mice were undetectable and consistent with the presence of high levels of the anti-inflammatory cytokine IL-10 in the serum at this time (Figure 1E). In the brain, genes encoding the cytokines IL-1β, IL-6, and TNF-α were similarly significantly induced within 3 h after a single dose of LPS (Figure 2). In contrast, expression of *Il1b*, *Il6*, and *Tnf* was significantly reduced after 4 consecutive daily doses of LPS when compared to mice given a single dose, and this was accompanied by an increase in the expression of *Il10* mRNA in the brains of 4xLPS-treated mice (Figure 2). Together, these data suggest that consecutive IP injections with LPS for 4 days induced innate immune tolerance in the periphery and brain, consistent with previous studies (Wendeln et al., 2018).

### Impact of Systemic Lipopolysaccharide Treatment on Glial Cells in the Brain

**FIGURE 3:**
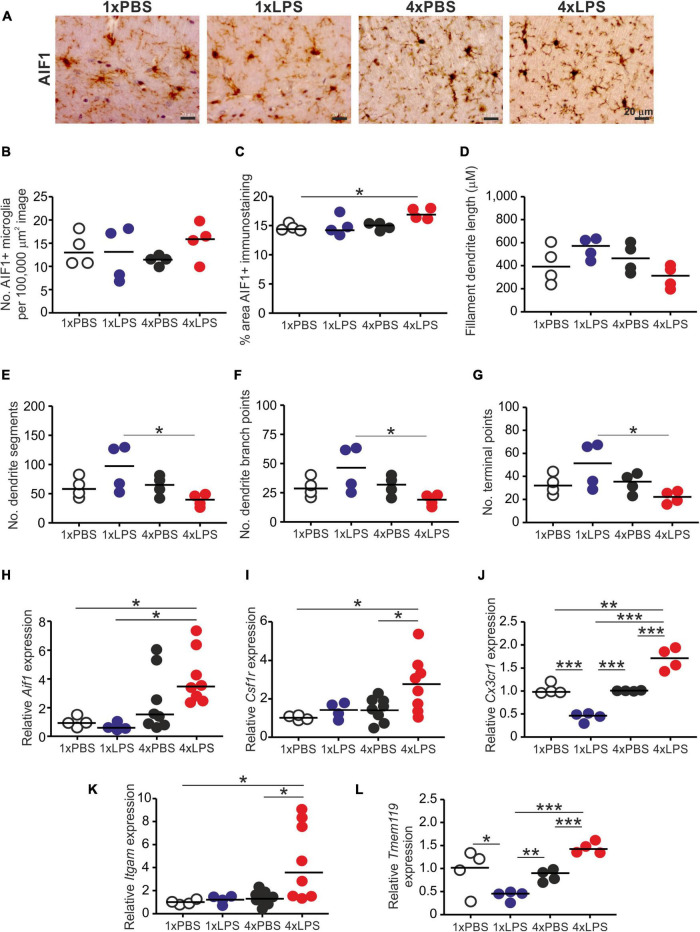
Effect of systemic LPS treatment on microglia. Mice were given a single (1x) four (4x) daily IP injections with either LPS or PBS (control) and brains collected 3 h after the final injection. **(A)** Representative immunostaining of AIF1 + microglia (brown) in the CA1 region of the hippocampus striatum radiatum of each mouse. Scale bar 20 μm. **(B)** Relative abundance of AIF1+ microglia in 100,000 μm^2^ images of the CA1 region of the hippocampus striatum radiatum of mice from each group. **(C)** Comparison of the magnitude of the AIF1+ immunostaining in the CA1 region of the hippocampus striatum radiatum of mice from each group. Assessment of AIF1+ microglia morphology in the CA1 region of the hippocampus striatum radiatum of mice from each group: **(D)**, mean filament dendrite length; **(E)**, mean number of dendrite segments; **(F)**, mean number of dendrite branch points; **(G)**, mean number of terminal points. **(H–L)** Relative expression level of *Aif1*, *Csf1r*, *Cx3cr1*, *Itgam*, and *Tmem119* mRNA in half brains from each mouse from each group. Gene expression data are normalized so that the mean level in the 1xPBS controls was 1.0. *N* = 4–8 mice/group; horizontal bar in histograms, median. *P < 0.05; **P < 0.01; ***P < 0.001.

Prion disease in the CNS is accompanied by extensive glial cell activation, and both the microglia and the astrocytes appear to play important roles in the development of the neuropathology (Zhu et al., 2016; Smith et al., 2020; Bradford et al., 2021). Since innate immune tolerance in microglia can influence the neuropathology in a mouse model of Alzheimer’s disease (Wendeln et al., 2018), we next compared the effects of a single and four daily LPS treatments on microglia and astrocytes. Brains were collected 3 h after the last LPS or PBS treatment, and microglia were detected on histological sections by immunostaining for allograft inflammatory factor-1 (AIF1+ cells; Figure 3A). Although LPS treatment did not affect the abundance of microglia in the brain when compared to PBS-treated controls (Figure 3B), a significant increase in the abundance of AIF1+ immunostaining was detected in the brains of 4xLPS-treated mice compared to PBS-treated controls (Figure 3C). Morphometric analysis suggested this was accompanied by a significant reduction in microglia dendrite branching morphology (number of dendrite segments, dendrite branch points, and terminal points) in the brains of 4xLPS-treated mice compared to the microglia in the brains of 1xLPS-treated mice (Figures 3D–G). Consistent with these changes, the expression of the microglia-related genes *Aif1*, *Csf1r*, *Cx3cr1* (encoding C-X3-C motif chemokine receptor 1), *Itgam* (encoding CD11b), and *Tmem119* (encoding transmembrane protein 119) were also significantly increased in the brains of 4xLPS-treated mice (Figures 3H–L). In the brains of mice given consecutive daily systemic LPS treatment (4xLPS-treated mice) the increased expression of the homeostatic microglia-related genes *Csf1r*, *Cx3cr1*, and *Tmem119*, elevated expression of mRNA encoding the anti-inflammatory cytokine IL-10, coupled with the reduced expression of the pro-inflammatory cytokines *Il1b*, *Il6* and *Tnf* implied that their microglia had a homeostatic/anti-inflammatory phenotype.

**FIGURE 4:**
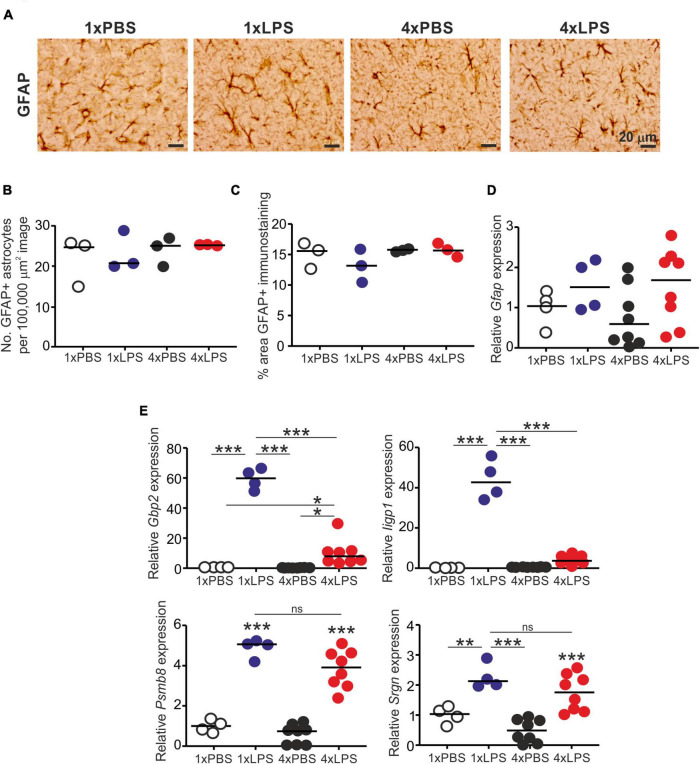
Effect of systemic LPS treatment on astrocytes. Mice were given a single (1x) or four (4x) daily IP injections with either LPS or PBS (control) and brains collected 3 h after the final injection. **(A)** Representative immunostaining of GFAP + astrocytes (brown) in the CA1 region of the hippocampus striatum radiatum of each mouse. Scale bar 20 μm. **(B)** Relative abundance of GFAP+ astrocytes in 100,000 μm^2^ images of the CA1 region of the hippocampus striatum radiatum of mice from each group. **(C)** Comparison of the magnitude of the GFAP+ immunostaining in the CA1 region of the hippocampus striatum radiatum of mice from each group. **(D)** Relative expression level of *Gfap* mRNA in half brains from each mouse from each group. **(E)** Relative expression level of *Gbp2*, *Iigp1*, *Psmb8*, and *Srgn* mRNA in half brains from each mouse from each group. Gene expression data are normalized so that the mean level in the 1xPBS controls was 1.0. *N* = 3–8 mice/group; horizontal bar in histograms, median. *P < 0.05; **P < 0.01; ***P < 0.001; ns, not significantly different.

Treatment with LPS did not affect the abundance of glial fibrillary acidic protein (GFAP)+ astrocytes (Figures 4A,B) or the abundance of GFAP protein or mRNA (Figures 4C,D). Factors including complement component C1q, IL-1α and TNF-α released from LPS-stimulated microglia can induce A1 neurotoxic astrocyte activation (Liddelow et al., 2017). Here, a single LPS treatment similarly induced the expression of the A1 neurotoxic reactive astrocyte-associated genes *Gbp2*, *Iigp1*, *Psmb8*, and *Srgn* in the brain (Figure 4E). Conversely, expression of *Gbp2* and *Iigp1* was significantly reduced in the brains of 4xLPS-treated mice (Figure 4E), implying that the innate immune tolerance induced by 4xLPS treatment had impeded the ability of the microglia to sustain A1 neurotoxic astrocyte activation. However, it was noticeable that expression of *Psmb8* and *Srgn* was not reduced in the brains of 4xLPS-treated mice when compared to 1xLPS-treated mice.

**FIGURE 5:**
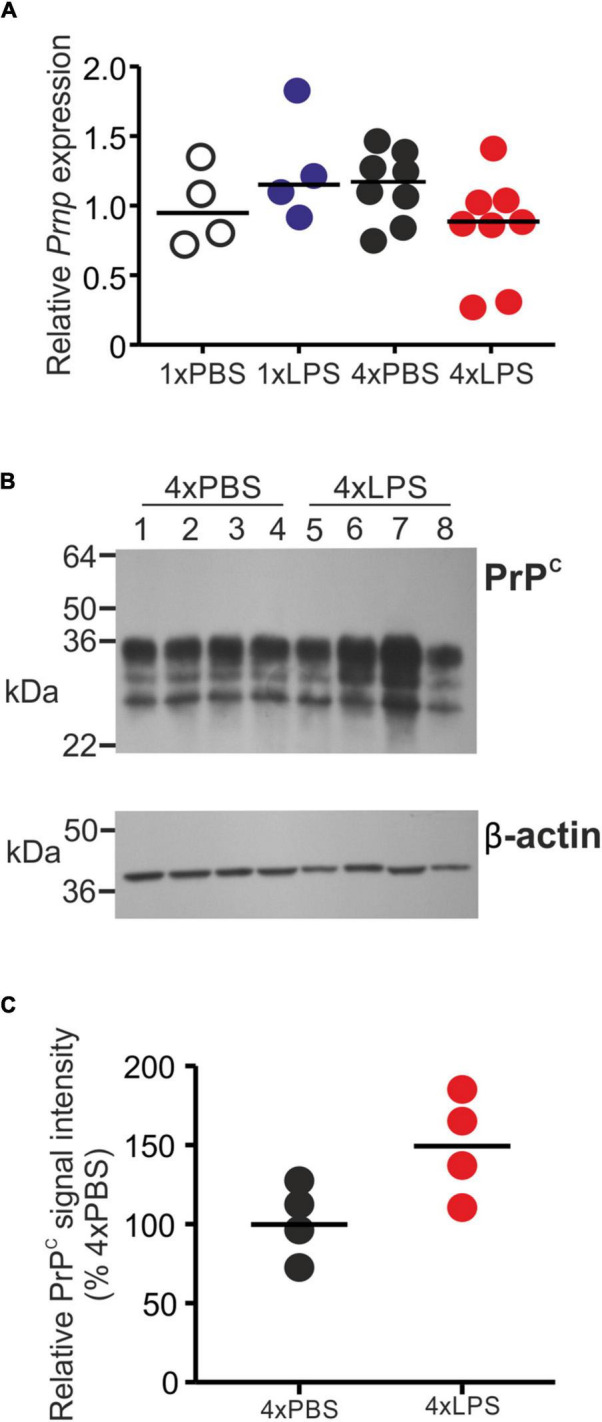
Effect of systemic LPS treatment on PrP^C^ expression in the brain. Mice were given a single (1x) or four (4x) daily IP injections with either LPS or PBS (control) and brains collected 3 h after the final injection. **(A)** Relative expression level of *Prnp* mRNA in half brains from each mouse from each group. Gene expression data are normalized so that the mean level in the 1xPBS controls was 1.0. *N* = 4–8 mice/group. Not significantly different. **(B)** Western blot analysis of PrP^C^ protein (upper panel) and β-actin (lower panel) in half brains from mice given 4 daily IP injections with either LPS or PBS (4xPBS, 4xLPS, respectively). **(C)** Quantitation of the relative abundance of PrP^C^ protein in half brains from each group. The abundance of PrP^C^ in each sample was normalized to β-actin and expressed as the% relative to the mean value in the 4xPBS-treated controls. *N* = 4 mice/group; horizontal bar in histogram, median. Not significantly different, student’s *t*-test.

The level of cellular PrP^C^ in the brain can indirectly influence the rate of development of CNS prion disease in infected mice (Manson et al., 1994). However, the expression levels of PrP^C^ mRNA (*Prnp*) and protein were similar in the brains of LPS-treated mice compared to PBS-treated controls (Figures 5A–C).

### Impact of Innate Immune Tolerance on Central Nervous System Prion Disease

Wendeln et al. (2018) showed how systemically induced innate immune tolerance causes epigenetic changes in the microglia that can influence the development of Alzheimer’s disease-like neuropathology in the brain several months after LPS treatment. We therefore determined whether the innate immune tolerance induced in the brains after four daily LPS treatments could similarly months later modify CNS prion disease pathogenesis.

**FIGURE 6:**
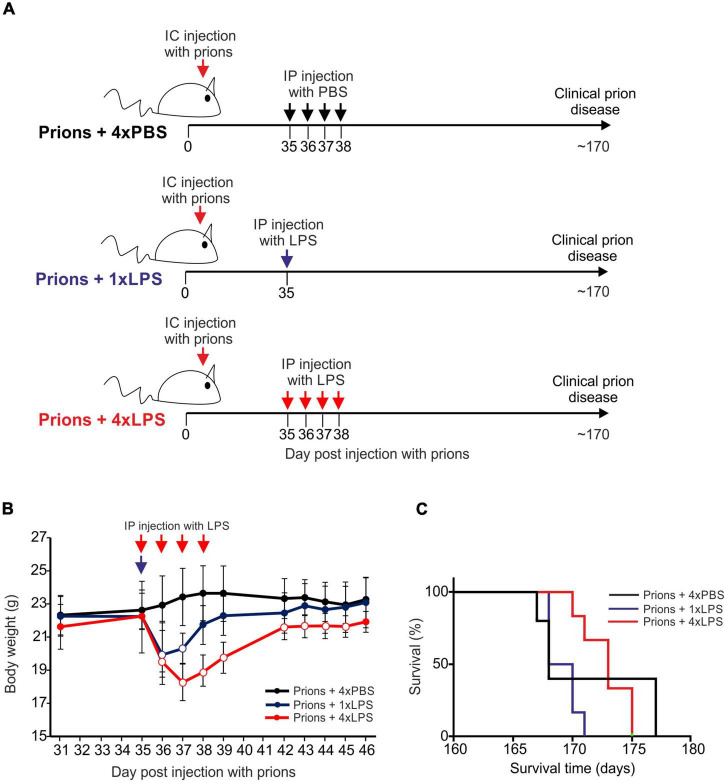
Effect of systemic LPS treatment on CNS prion disease. **(A)** Cartoon describing the experimental design. Mice were first injected with ME7 scrapie prions directly into the brain by IC injection. Thirty-five days later, the mice were given either a single IP LPS injection (prions + 1xLPS) or four consecutive daily IP LPS injections (prions + 4xLPS) to induce innate immune training or tolerance, respectively. A parallel group of mice were given four consecutive daily IP PBS injections (prions + 4xLPS) as a control. **(B)** A single IP LPS injection (prions + 1xLPS) or four consecutive daily IP LPS injections (prions + 4xLPS) caused a transient reduction in body weight compared to prions + 4xPBS-treated controls. Open circles, *P* < 0.05 compared to prions + 4xPBS-treated controls. Closed circles, not significantly different compared to prions + 4xPBS-treated controls. **(C)** Survival curve for prion infected mice in each treatment group. *N* = 5–6 mice/group. Not significantly different, Log-rank (Mantel-Cox) test.

Groups of female C57BL/6J mice (*n* = 5–6/group) were first injected with ME7 scrapie prions directly into the brain by intracerebral (IC) injection. At 35 days post injection (dpi) with prions, and several weeks prior to the onset of detectable histopathological signs of microglia activation and prion-specific neuropathology in the brain (Donaldson et al., 2020; Bradford et al., 2021), the mice were then given a single IP LPS injection (prions + 1xLPS) or four consecutive daily IP LPS injections (prions + 4xLPS) to induce innate immune training or tolerance, respectively, as above (Figure 6A). A third group of mice were treated with PBS as a control (prions + 4xPBS). The mice were then monitored for the development of clinical signs of prion disease.

As above, peripheral LPS treatment caused a transient period of mild sickness behavior and weight loss when compared to the PBS-treated mice which had resolved by 7 days later (Figure 6B). All prion-infected PBS-treated control mice succumbed to clinical prion disease with a mean survival time of 172 ± 3 days (Figure 6C and Table 1). Treatment with a single or four consecutive daily doses of LPS did not significantly affect prion disease survival times. All the mice from the prions + 1xLPS and prions + 4xLPS treatment groups succumbed to clinical prion disease with similar survival times when compared to the prions + 4xPBS control mice (Figure 6C and Table 2). The timing of the duration to the initial onset of detection of the clinical signs of prion disease and the duration of the clinical phase were also similar in each group of mice irrespective of treatment (Table 2). These data clearly demonstrate that the innate immune tolerance induced after four daily LPS treatments did not affect the subsequent onset of the development of the clinical signs of prion disease.

**TABLE 2:** Effect of systemic LPS treatment on CNS prion disease.

Group	Incubation period^a^	Clinical phase^b^	Survival time^C^	Clinical disease incidence^d^
Prions + 4xPBS	159 ± 7 (160)	12 ± 5 (10)	171 ± 5 (168)	5/5
Prions + 1xLPS	164 ± 3 (167)ns	6 ± 5 (4)ns	169 ± 1 (169)ns	6/6
Prions + 4xLPS	165 ± 4 (167)ns	6 ± 4 (4)ns	173 ± 2 (173)ns	6/6

*Mice were first injected with ME7 scrapie prions directly into the brain by IC injection. Thirty five days later the mice were given a either single IP LPS injection (Prions + 1xLPS) or four consecutive daily IP LPS injections (Prions + 4xLPS) to induce innate immune training or tolerance, respectively. A parallel group of mice were given four consecutive daily IP PBS injections (Prions + 4xLPS) as a control.*

*^a^Incubation period, mean duration (days ± SD) from time of IC injection with prions to time when first clinical signs of disease were detected. Median duration in brackets.*

*^b^Clinical phase, mean duration (days ± SD) from first detection of clinical signs of prion disease to development of terminal signs of clinical disease. Median duration in brackets.*

*^c^Survival time, mean duration (days ± SD) from time of IC injection with prions to development of terminal signs of clinical disease. Median duration in brackets.*

*^d^Clinical disease incidence, number of mice that developed terminal clinical signs of prion disease/number of mice injected IC with prions.*

*Statistical differences between groups were compared using One-Way ANOVA and post hoc Tukey multiple comparisons tests. Survival data compared by Log-rank (Mantel-Cox) test. ns, not significant.*

**FIGURE 7:**
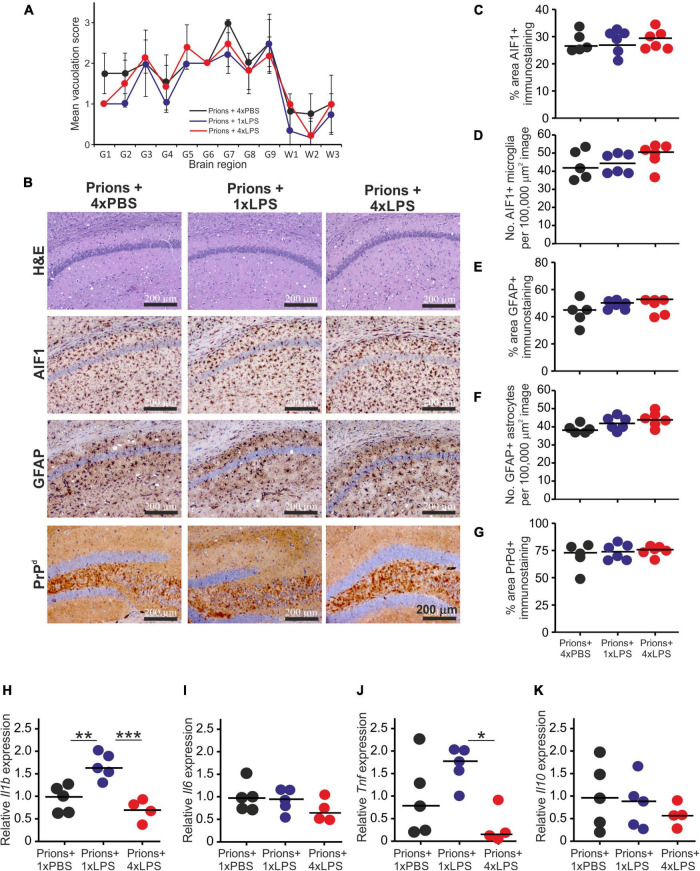
Effect of systemic LPS treatment on the histopathological signs of prion disease in the brain. Mice were first injected with ME7 scrapie prions directly into the brain by IC injection. Thirty five days later the mice were given a either single IP LPS injection (prions + 1xLPS) or four consecutive daily IP LPS injections (prions + 4xLPS) to induce innate immune training or tolerance, respectively. A parallel group of mice were given four consecutive daily IP PBS injections (prions + 4xLPS) as a control. Brains were collected at the terminal stage. **(A)** The severity and distribution of the spongiform pathology (vacuolation) within each clinically affected brain from each treatment group was scored on H&E sections using a scale of 1–5 in nine gray matter regions and three white matter regions: G1, dorsal medulla; G2, cerebellar cortex; G3, superior colliculus; G4, hypothalamus; G5, thalamus; G6,hippocampus; G7, septum; G8, retrosplenial and adjacent motor cortex; G9, cingulate and adjacent motor cortex; W1, inferior and middle cerebellar peduncles; W2, decussation of superior cerebellar peduncles; and W3, cerebellar peduncles. **(B)** Representative images showing high levels of spongiform pathology (H&E, upper row), microgliosis (AIF1+ cells, brown, 2nd row), reactive astrocytes (GFAP+ cells, brown, 3rd row) and heavy prion disease-specific PrPd accumulation (brown, bottom row) in the hippocampus of all prion infected mice from each treatment group at the terminal clinical stage. Sections counterstained with hematoxylin to detect cell nuclei (blue). Scale bar, 200 μm. **(C,D)** Comparison of the magnitude of the AIF1+ immunostaining and abundance of AIF1 + microglia, respectively, in the CA1 region of the hippocampus of mice from each group. **(E,F)** Comparison of the magnitude of the GFAP+ immunostaining and abundance of GFAP+ reactive astrocytes, respectively, in the CA1 region of the hippocampus of mice from each group. **(G)** Comparison of the magnitude of the PrP*^d^* + immunostaining in the CA1 region of the hippocampus of mice from each group. **(H)** Relative expression level of *Il1b* mRNA. **(I)** Relative expression level of *Il6* mRNA. **(J)** Relative expression level of *Tnf* mRNA. **(K)** Relative expression level of *Il10* mRNA. Gene expression data are normalized so that the mean level in the 1xPBS controls was 1.0. *N* = 4–6 mice/group; horizontal bar, median. *P < 0.05; **P < 0.01; ***P < 0.001.

Histopathological analysis of the brains from each group of clinically affected mice showed that the magnitude and distribution of the prion disease-specific vacuolation were similar in clinically affected prions + 1xLPS and prions + 4xLPS treated mice compared to prions + 4xPBS controls (Figure 7A). The magnitude of the microgliosis and reactive astrocytosis in response to CNS prion infection was also similar in the brains of mice from each group (Figures 7B–F). We also compared the levels of mRNA encoding the cytokines IL-1β, IL-6, TNF-α and IL-10 in the brains of mice from each group. Innate immune tolerance induced after four daily LPS treatments did not affect the expression level of mRNA encoding these cytokines at the terminal stage of prion disease when compared to prions + 4xPBS controls (Figures 7H–K). However, microglia training in response to 1xLPS treatment coincided with a significant increase in *Il1b* expression at the terminal stage compared to prions + 4xPBS controls, and this increase was ablated in the brains of prions + 4xLPS treated mice (Figure 7H). The level of *Tnf* expression in the brains of prions + 4xLPS treated mice was also significantly reduced compared to prions + 1xLPS treated mice (Figure 7J).

**FIGURE 8:**
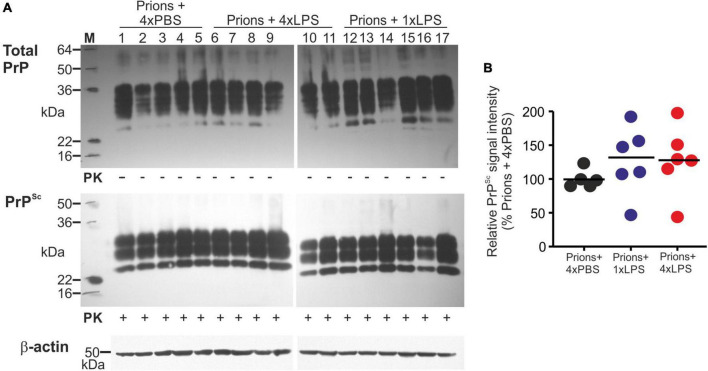
Effect of systemic LPS treatment on prion disease-specific PrP^Sc^ accumulation in the brain. Mice were first injected with ME7 scrapie prions directly into the brain by IC injection. Thirty five days later the mice were given a either single IP LPS injection (prions + 1xLPS) or four consecutive daily IP LPS injections (prions + 4xLPS) to induce innate immune training or tolerance, respectively. A parallel group of mice were given four consecutive daily IP PBS injections (prions + 4xLPS) as a control. Brains were collected at the terminal stage. **(A)** Western blot analysis of prion disease specific PrP^Sc^ accumulation in half brains from each group. PK, proteinase-K. Upper panel, total PrP; middle panel, relatively PK-resistant prion disease-specific PrP^Sc^; lower panel β-actin. **(B)** Quantitation of the relative abundance of PrP^Sc^ in half brains from each group. The abundance of PrP^Sc^ in each sample was expressed as the% relative to the mean value in the 1xPBS-treated controls. *N* = 5–6 mice/group; horizontal bar in histograms, median.

A previous study demonstrated that 4 consecutive LPS treatments induced long-term modifications in the brain that months later were sufficient to decrease amyloid-β levels and plaque burdens in the APP23 mouse model of Alzheimer’s disease pathology (Wendeln et al., 2018). However, LPS treatment did not subsequently affect the accumulation of prion disease-specific PrP^Sc^ accumulation in the brain at the terminal clinical stage (Figures 7B,G, 8A,B). Together, these data clearly demonstrate that innate immune tolerance induced by consecutive peripheral LPS treatments does not induce long-term modifications that significantly affect the development of CNS prion disease pathogenesis.

## Discussion

The microglia are considered to provide neuroprotection during CNS prion disease (Zhu et al., 2016; Bradford et al., 2021), but their pro-inflammatory activation may exacerbate the development of the neuropathology in affected regions of the brain (Cunningham et al., 2005; Cunningham et al., 2009; Alibhai et al., 2016). Innate immune tolerance induced by consecutive systemic LPS treatment causes long-term epigenetic changes in the microglia in the brain that several months later can impede the accumulation of insoluble amyloid-β and plaque-associated neuritic damage in a transgenic mouse model of Alzheimer’s disease-like pathology (Wendeln et al., 2018). We therefore reasoned that innate immune tolerance in microglia might similarly impede the subsequent development of CNS prion disease. However, in the current study, we clearly show that consecutive systemic LPS treatment did not affect the subsequent development of the neuropathology or survival times in a mouse model of CNS prion disease. Thus, whereas innate immune tolerance and the epigenetic modifications it causes in the microglia may influence the severity of the neuropathology of some neurological diseases including Alzheimer’s disease and cerebral ischemia (Wendeln et al., 2018), our data suggest it is unlikely to impact on the development of CNS prion disease.

During the 1980s the UK human population is estimated to have been extensively exposed to bovine spongiform encephalopathy (BSE) prions via contaminated food (Wilesmith, 1993; Valleron et al., 2001). Despite this widespread risk of zoonotic disease transmission, the numbers of definite and probable human cases of variant Creutzfeldt-Jakob disease (vCJD) due to the consumption of BSE-contaminated food have remained low (*n* = 178, March 2022), with no new cases recorded since 2016 (Unit, 2018). However, a higher incidence of prion disease-specific PrP accumulation has been detected in a retrospective screen of UK human appendix and tonsil samples (Hilton et al., 2004; Gill et al., 2013), raising the possibility that there may be many more asymptomatic individuals infected with vCJD. The reasons for the discrepancy between the reported number of probable and definite clinical vCJD cases and the estimated incidence of infected individuals in the UK are not known. Of course since these studies relied on the detection of disease-specific PrP by immunohistochemistry (IHC), it remains to be determined whether this reflects the presence of infectious vCJD prions (Gill et al., 2013). In the current study, we considered it plausible that systemic inflammation several months, or even years, before onset of the prion accumulation in the brain might cause innate immune tolerance that reduces the risk of subsequently developing CNS prion disease in sub-clinically infected individuals. However, using an experimental mouse model of CNS prion disease our data suggest that innate immune tolerance in the microglia is unlikely to have a significant impact on CNS prion disease susceptibility or the development of the neuropathology.

CNS prion infection has been suggested to induce a “primed state” in the microglia that exacerbates their subsequent responses to pro-inflammatory stimuli (Perry et al., 2003). As a consequence, a range of studies have shown how exposure to pro-inflammatory stimuli or a systemic infection during the CNS phase can switch the microglia from an anti-inflammatory phenotype to an activated disease-associated phenotype that can exacerbate the clinical signs and accelerate prion disease progression (Cunningham et al., 2005; Cunningham et al., 2009; Lunnon et al., 2011; Chouhan et al., 2022). In contrast, in the current study, the mice were treated with LPS several months before the induction of the primed state in the microglia and the onset of the neuropathology. Further studies are necessary to determine whether prolonged LPS exposure during the CNS phase would similarly impact on the development of the neuropathology, for example by impeding microglia polarization toward a disease-associated phenotype (Alibhai et al., 2016).

Whereas Wendeln et al. (2018) showed how the long-term effects of innate immune tolerance in the microglia impeded the development of amyloid-β-associated neuropathology in a transgenic mouse model of Alzheimer’s disease-like pathology, our data show this did not affect the development of CNS prion disease pathology. These contrasting effects highlight important differences in the phenotypes of the microglia in the prion disease-affected brain when compared to other neurodegenerative disorders. For example, expression of triggering receptor expressed on myeloid cells-2 (TREM2) is important for the disease-associated activation of the microglia in a mouse model of Alzheimer’s disease-like pathology (Keren-Shaul et al., 2017). In contrast, TREM2 deficiency has only a modest influence on CNS prion disease (Zhu et al., 2015). The absence of the commensal gut or lung microbiota can significantly affect microglial activation (Erny et al., 2015) and the development of autoimmunity in the CNS (Hosang et al., 2022), but we have shown that CNS prion disease was unaltered in the germ-free mice (Bradford et al., 2017b). Factors produced by the microglia can trigger neurotoxic A1 reactive astrocyte activation (Liddelow et al., 2017). However, the reactive astrocyte activation in the prion disease-affected brain occurs independently of stimulation from the microglia or microglia-derived factors (Hartmann et al., 2020; Bradford et al., 2021). Instead, during prion disease, the microglia appear to be acting to constrain the neurotoxic or dysregulated activation of reactive astrocytes (Bradford et al., 2021).

No therapies or cures are currently available to treat the prion diseases. The pharmacological modulation of microglia phenotype or activation status has been proposed a novel means to prevent or delay the development of the neuropathology (Zhu et al., 2016; Bradford et al., 2021; Hu et al., 2021). Our data clearly suggest that innate immune tolerance in the microglia is unlikely to have a significant impact on CNS prion disease. However, a thorough understanding of the impact of systemic inflammation on microglia during CNS prion disease could help identify important factors that influence the risk of developing prion disease and other important neurodegenerative conditions.

## Data Availability Statement

The original contributions presented in this study are included in the article/supplementary material, further inquiries can be directed to the corresponding author/s.

## Ethics Statement

The animal studies were reviewed and approved by the University of Edinburgh’s Ethical Review Committee.

## Author Contributions

NAM conceived the study and obtained funding. RP and BMB performed the experiments and analyzed the datasets. All authors designed the study, contributed to the writing of the manuscript, and approved the final draft.

## Conflict of Interest

The authors declare that the research was conducted in the absence of any commercial or financial relationships that could be construed as a potential conflict of interest.

## Publisher’s Note

All claims expressed in this article are solely those of the authors and do not necessarily represent those of their affiliated organizations, or those of the publisher, the editors and the reviewers. Any product that may be evaluated in this article, or claim that may be made by its manufacturer, is not guaranteed or endorsed by the publisher.

## Footnotes

1
https://imagej.nih.gov/ij/
